# Comparative analysis of silencing expression of myostatin (*MSTN*) and its two receptors (*ACVR2A* and *ACVR2B*) genes affecting growth traits in knock down chicken

**DOI:** 10.1038/s41598-019-44217-z

**Published:** 2019-05-24

**Authors:** T. K. Bhattacharya, Renu Shukla, R. N. Chatterjee, S. K. Bhanja

**Affiliations:** ICAR-Directorate of Poultry Research, Rajendranagar, Hyderabad, India

**Keywords:** Genetic engineering, RNAi

## Abstract

Myostatin (MSTN), a growth differentiation factor-8 regulates muscular development through its receptors, ACVR2A (Activin receptor type IIA) and ACVR2B (Activin receptor type IIB) by inhibiting cellular differentiation of developing somites during embryonic stage and diminishing myofibriller growth during post-embryonic period. The objective of this study was to compare the effect of knockdown of expression of myostatin, *ACVR2A* and *ACVR2B* genes on growth traits in chicken. The shRNAs for Myostatin, *ACVR2A* and *ACVR2B* genes were designed, synthesized and cloned in DEST vector. The recombinant molecules were transfected into the spermatozoa and transfected spermatozoa were inseminated artificially to the hens to obtain fertile eggs. The fertile eggs were collected, incubated in the incubator and hatched to chicks. Silencing of *ACVR2B* gene showed significantly higher body weight than other single, double and triple knock down of genes in transgenic birds. The carcass traits such as dressing%, leg muscle%, and breast muscle% were found with the highest magnitudes in birds with silencing of the *ACVR2B* gene as compared to the birds with that of other genes and control group. The lowest serum cholesterol and HDL content was found in *ACVR2B* silencing birds. The total RBC count was the highest in this group though the differential counts did not differ significantly among various silencing and control groups of birds. It is concluded that silencing of only one receptor of *MSTN* particularly, *ACVR2B* may augment the highest growth in chicken during juvenile stage. Our findings may be used as model for improving growth in other food animals and repairing muscular degenerative disorders in human and other animals.

## Introduction

Myostatin, a growth differentiating factor-8 is a member of transforming growth factor-β family expressed predominantly in the muscle tissues. Myostatin inhibits muscular development through cellular differentiation of developing somites during embryonic stage and growth of myofibrillar cells during adult stages in animals^1,2^. Thus, myostatin (MSTN) is regarded as the typical negative regulator of myogenesis in animals^3^. Earlier investigations have demonstrated biological activity of MSTN through its receptors such as activin receptor type IIB (ACVR2B, also known as ActrIIB) and activin receptor type IIA (also known as ACVR2A or ActrIIA). Both the receptors are also the members of TGF-ß super-family^4^. It is known that improper biological function of MSTN on account of mutation in the gene has been associated with the double muscling phenotype in cattle, suggesting that *MSTN* gene normally functions as a negative regulator of skeletal muscle growth in animals^5^. The function of MSTN also determined to be conserved across the species, as animals with genetic mutations in the *MSTN* gene, found in Belgian blue cattle, mouse, the whippet dog and human exhibiting hyper muscled phenotype^6–10^. In mice, absence of myostatin gene showed an enormous increase in skeletal muscle mass, which made animals approximately twice as those of wild type mice on account of muscle fiber hyperplasia and hypertrophy^11^. The enhanced expression of myostatin gene was found in humans having significant association with chronic illness, HIV infection and early aging due to muscle atrophy^12–14^. Thus, the MSTN has been a prime target for the development of therapies for chronic muscle degeneration (such as sarcopenia or muscle degenerative diseases), acute muscle loss (such as cachexia), and even metabolic diseases (such as obesity and Type II diabetes) in human. The discovery of biological inhibitors of MSTN, such as follistatin^7^, follistatin-related gene^15^, GDF associated serum protein-1^16^, *MSTN* propeptide^15^, *MSTN* receptors^17,18^, ALK4 and ALK5^19^ have offered a multifaceted approach for the treatment of muscular degenerative diseases through the neutralization of *MSTN* in the circulation. Exploiting these naturally occurring inhibitors or their derivatives by means of overexpression^20^ or gene delivery^16^ has produced substantial improvements in muscle mass, which implies that the regulation of skeletal muscle is not the sole responsibility of *MSTN* but is shared by other members of the transforming growth factor-β superfamily.

Activin IIA receptor mRNAs was first detected by *in situ* hybridization in embryonic spinal cord and ciliary ganglion neurons in chicken^21,22^. The mRNAs have also been reported to be present in the dorsal root ganglia during embryonic period, day 12–20 in rat and 12.5 day in mouse^23,24^. The extracellular domains of *ACVR2A* contained a three-fingered toxin fold^25,26^, which was formed by three pairs of anti-parallel β-sheets, beta1– beta2, beta3– beta4 and beta5–beta6^25,26^. The cytoplasmic domain of ACVR2A is highly conserved and has the kinase activity. The protein has a small N-terminal lobe containing a five stranded anti-parallel β-sheet and a single α-helix, while a large C-terminal lobe containing α-helix and a loop involved in polypeptide binding. The N- and C-terminal lobes are connected by a hinge sequence, encompassing the binding site for ATP. The *ACVR2A* can be activated not only by activins, but also by other ligands, including myostatin and bone morphogenic proteins (BMPs). Thus, the ACVR2A is involved in a variety of biological functions including muscle growth, bone formation and viability as well as adhesion of prostatic epithelial cells^27^.

The activin type IIB receptor is a transmembrane serine-threonine kinase receptor for many members of the transforming growth factor-β (TGF) superfamily involved in the negative regulation of growth of muscle tissues through GDF8 pathway^28^. Thus, pharmacological capability of ACVR2B by blocking MSTN signaling pathway may have applications for promoting muscle growth in livestock/animals.

Rapid improvement of muscling in meat producing animals through lowering expression of *MSTN* and its receptors may be one of the most important approaches in livestock and poultry industry. The lowering expression of genes can be possible through many approaches of which gene silencing by shRNA has been one of them. In gene silencing, genomic modification of gene is not done but its transcribed products are degraded upon cleaving by shRNA molecules. In case of stable transfection, shRNA molecules are synthesized continuously, which has been a popular technique to achieve sequence-specific knockdown of target mRNA. Ability to obtain potent and stable RNAi silencing is critical for number of applications especially for gene knockdown of target gene validation^29^, functional genomic analysis^30^ and therapeutic use^31^. This approach in poultry may also be a role model for treatment of muscle decaying diseases in human. Hence, the objectives of the study were to silence the expression of *MSTN* and its receptors genes and to analyse its relative impact on growth and associated traits in transgenic chicken.

## Results

### Knock-down chicken

**Figure 1:**
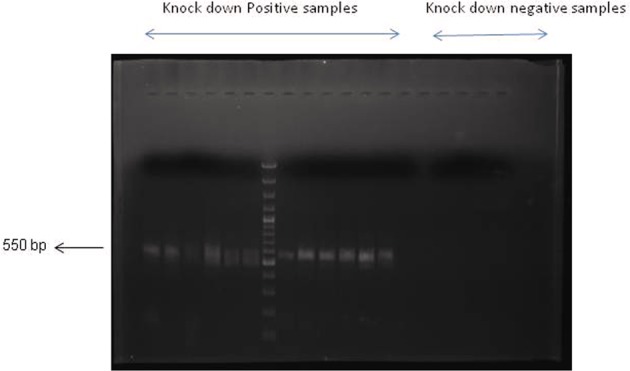
PCR based screening of knock down birds where presence of 550 bp fragment reveals the presence of recombinant DEST vector present in the genome.

**Figure 2:**
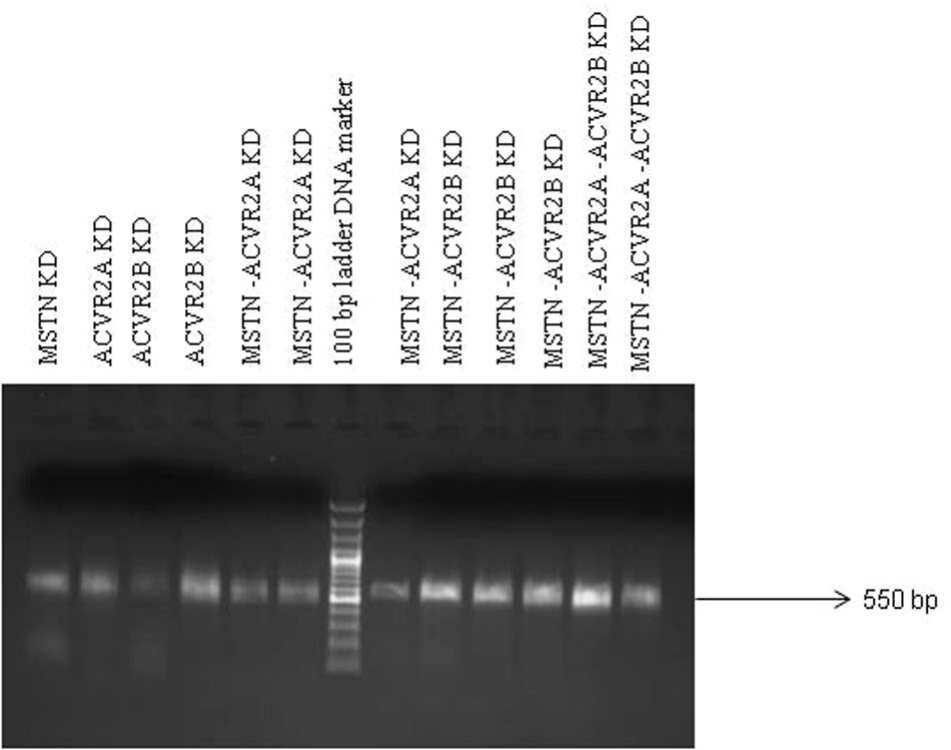
PCR based screening of knock down birds where presence of 550 bp fragment reveals the presence of recombinant DEST vector present in the genome. KD denotes knock down.

**Figure 3:**
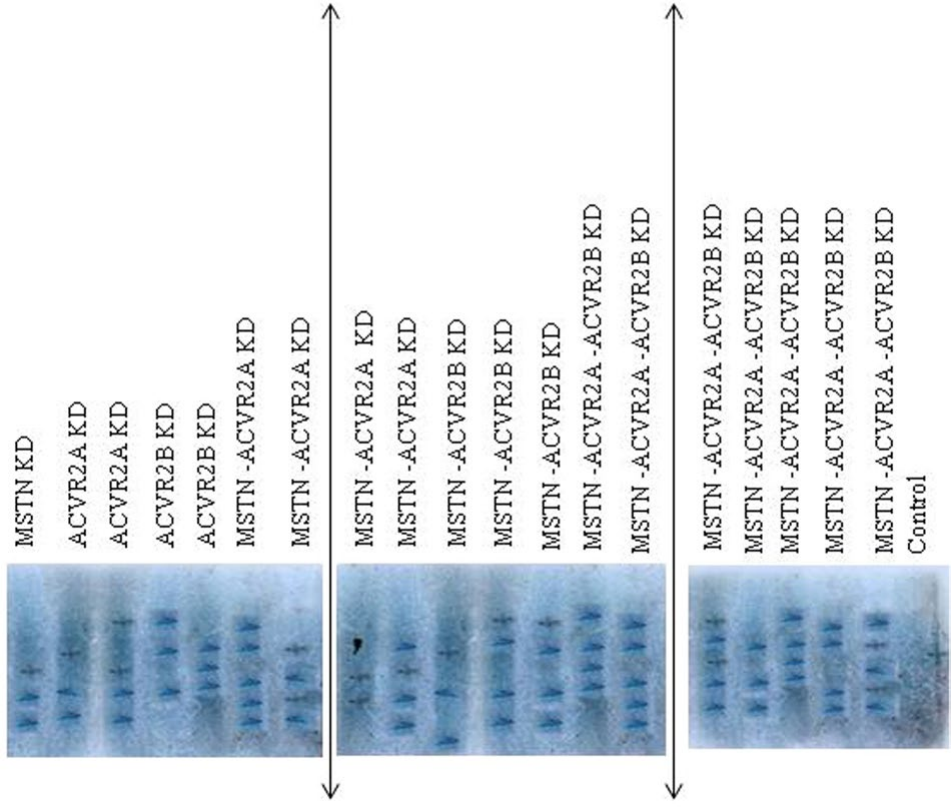
Southern blot of *Pst*I digested genomic DNA collected from knock down and control chicken. KD denotes knock down. Blots are separated by black lines.

**Figure 4:**
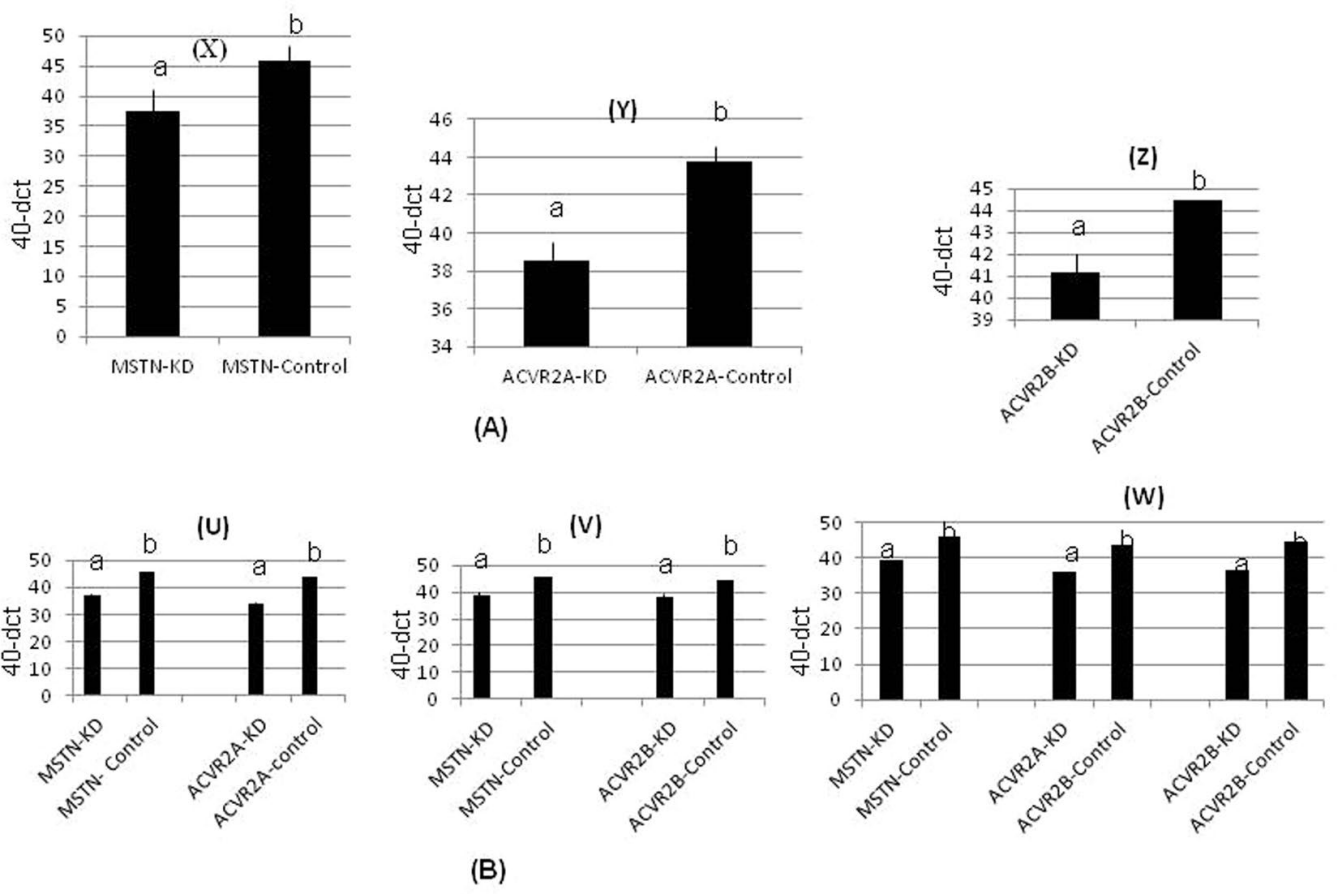
(**A**) The mRNA expression level in knock down and control birds. (X) *MSTN* knock down group where *MSTN*-KD denotes level of *MSTN* mRNA in *MSTN* knock down birds and *MSTN*-Control denotes level of *MSTN* mRNA in control birds. (Y) ACVR2A knock down group where ACVR2A-KD denotes level of ACVR2A mRNA in ACVR2A knock down birds and ACVR2A-Control denotes level of ACVR2A mRNA in control birds. (Z) *ACVR2B* knock down group where *ACVR2B*-KD denotes level of *ACVR2B* mRNA in *ACVR2B* knock down birds and *ACVR2B*-Control denotes level of *ACVR2B* mRNA in control birds. Different superscripts indicate significance at P < 0.05. (**B**) The mRNA expression level in knock down and control birds. (U) Double knock down (*MSTN* & ACVR2A) group where *MSTN*-KD denotes level of *MSTN* mRNA in double knock down birds; *MSTN*-Control denotes level of *MSTN* mRNA in control birds; ACVR2A-KD denotes level of ACVR2A mRNA in double knock down birds and ACVR2A-Control denotes level of ACVR2A mRNA in control birds. (V) Double knock down (*MSTN* & *ACVR2B*) group where *MSTN*-KD denotes level of *MSTN* mRNA in double knock down birds; *MSTN*-Control denotes level of *MSTN* mRNA in control birds; *ACVR2B*-KD denotes level of *ACVR2B* mRNA in double knock down birds and *ACVR2B*-Control denotes level of *ACVR2B* mRNA in control birds. (W) Tripple knock down (*MSTN*, ACVR2A & *ACVR2B*) group where *MSTN*-KD denotes level of *MSTN* mRNA in tripple knock down birds; *MSTN*-Control denotes level of *MSTN* mRNA in control birds, ACVR2A-KD denotes level of ACVR2A mRNA in tripple knock down birds and ACVR2A-Control denotes level of ACVR2A mRNA in control birds; *ACVR2B*-KD denotes level of *ACVR2B* mRNA in tripple knock down birds and *ACVR2B*-Control denotes level of *ACVR2B* mRNA in control birds. Different superscripts indicate significance at P < 0.05.

**Figure 5:**
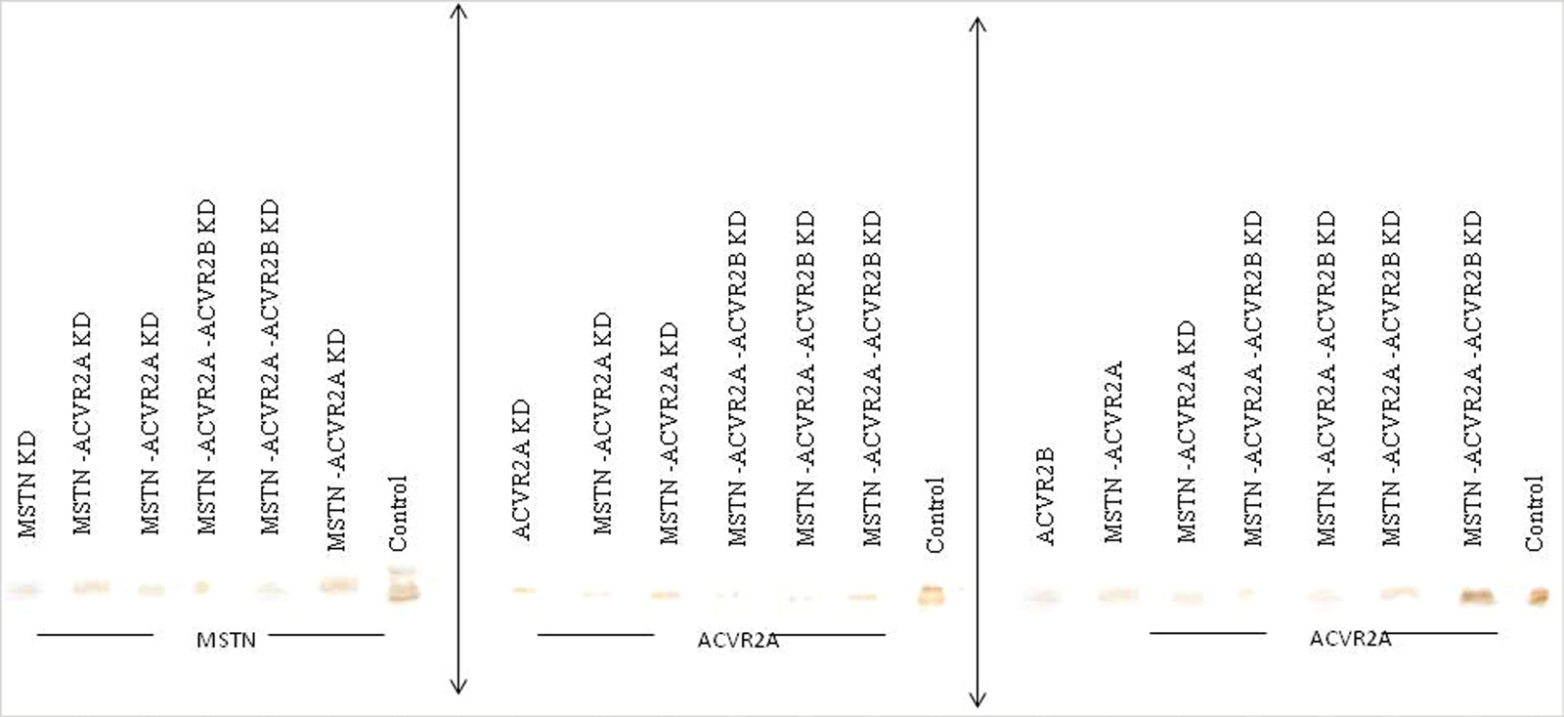
Western blot of myostatin/ACVR2A/*ACVR2B* expressed in breast muscle tissue collected from knock down and control groups of chicken. Blots are separated by black lines. KD denotes knock down.

**Figure 6:**
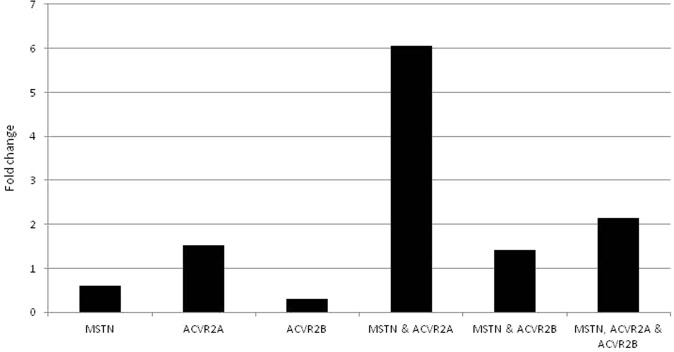
The fold change of mRNA expression of IFN-gamma in control group as compared to knock down birds. The expression was not differed significantly among the groups.

Sperm mediated gene transfer method (SMGT) was employed to develop knock down chicken. The hatching percentage varied among the treatment groups ranging from 60% in *MSTN*-*ACVR2A*-*ACVR2B* group to 75% in *ACVR2B* group (Table 1). All the birds were screened with PCR (Figs 1 and 2) and Southern blot (Fig. 3). The success rate of obtaining positive transgenic birds ranged from 6.8% in case of *MSTN* to 66.6% in *MSTN*-*ACVR2A*-*ACVR2B* group. After chicks were hatched, some of the chicks were died without any anatomical or other defects. The death rate was found to be the highest in *MSTN*-*ACVR2A*-*ACVR2B* group whereas the lowest rate (0%) was found in *MSTN* group. The expression of *MSTN*, *ACVR2A* and *ACVR2B* genes in transgenic group was detected by real time PCR, Western blotting and ELISA. The control group of birds showed higher mRNA expression of target genes over the single, double and triple knock down birds (Fig. 4A,B). In terms of fold change of expression, the control birds showed 337 folds higher expression of myostatin mRNA in myostatin knock down birds while the control birds had 831 and 222 folds higher expression of *ACVR2A* and *ACVR2B* mRNA than *ACVR2A* knock down and *ACVR2B* knock down birds, respectively. In double knock down birds, the control birds showed 512 and 776 folds higher expression of myostatin and *ACVR2A* mRNAs, respectively over the *MSTN* and *ACVR2A* double knock down birds. Likewise, the control birds showed 168 and 81 folds higher expression of *MSTN* and *ACVR2B* mRNAs, respectively over the *MSTN* and *ACVR2B* double knock down birds. In case of triple knock down, the control birds showed 93, 194 and 238 folds higher expression of *MSTN*, *ACVR2A* and *ACVR2B* mRNAs, respectively over the *MSTN*, *ACVR2A* and *ACVR2B* triple knock down birds. The Western blotting revealed the expression of the proteins in both knock down and control birds (Fig. 5). The ELISA results mentioned in the Table 1S also corroborated the lower levels of MSTN, ACVR2A and ACVR2B proteins in serum of single, double and triple knock down birds over the control group of birds. Knocking down of MSTN, ACVR2A and ACVR2B genes in the knock down birds reduced the level of mRNA expressions in the tissues, which lead to lower level of protein expression. The higher titres for MSTN, ACVR2A and ACVR2B were observed in the control group as compared to the knock down groups indicating higher serum concentrations of MSTN/ACVR2A/ACVR2B in the control group of birds. Thus, between knock down and control groups, the serum MSTN/ACVR2A/ACVR2B concentrations were diminished at the knock down birds as compared to the control groups indicating efficiency of knock down experiment to lower the level of MSTN/ACVR2A/ACVR2B protein expression. Further, we have also analysed the concurrent expression of interferon gamma gene in the knock down birds to explore the impact of shRNA on immune function. In our study, the introduction of recombinant shRNA clones did not show significant effect (P < 0.05) on expression of interferon gamma gene in knock down birds, which revealed the efficiency of shRNA molecules in development of knock down chicken (Fig. 6).

**Table 1:** Hatching profile of knock down and control chicks.

Knock down gene (s)	Number eggs incubated	Chicks hatched	Hatching%	shRNA positive chicks	Percentage of positive birds	Death percentage of positive birds
*MSTN*	40	29	72.5	2	6.8	−
ACVR2A	40	27	67.5	6	22.2	16.6
*ACVR2B*	40	30	75.0	14	46.6	7.1
*MSTN*-*ACVR2A*	40	28	70.0	16	57.1	12.5
*MSTN*-*ACVR2B*	40	25	62.5	11	44.0	9.0
*MSTN*-*ACVR2A*- *ACVR2B*	40	24	60.0	16	66.6	37.5
Control	40	29	72.5	−	−	−

### Effect of knock down on growth traits

The silencing of myostatin and its two receptors namely, *ACVR2A* and *ACVR2B* significantly (P < 0.05) affected body weights at different ages in chicken (Table 2). At day old age, silencing of *MSTN* gene showed the highest body weight in chicken. At day 28, silencing of only *ACVR2B* and *MSTN*, and combination of *MSTN*, *ACVR2A* and *ACVR2B* showed higher body weights than the control birds. However, silencing of *ACVR2B* gene in transgenic birds had the highest body weight at 28^th^ (454.2 ± 18.7 g), 35^th^ (703.4 ± 20.6 g) and 42^nd^ (779.1 ± 21.4 g) day of age. In case of *MSTN* knock down and triple knock down of *MSTN*, *ACVR2A* and *ACVR2B* genes, respectively, the body weight at 35^th^ day (668.3 ± 12.2 and 657.2 ± 32.4 g) and 42^nd^ day (735.9 ± 14.0 and 732.1 ± 32.8 g) was significantly higher than the control group (581.5 ± 8.8 and 639.1 ± 10.3 g).

**Table 2:** Growth traits in terms of body weights in knock down and control chicken. Column-wise different superscripts indicate significance at P < 0.05.

Knock down gene (s)	Average body weight (g) at				
	Day old	2^nd^ week	4^th^ week	5^th^ week	6^th^ week
*MSTN*	45.9 ± 2.5^b^	158.9 ± 0.5	450.1 ± 28.7^b^	668.3 ± 12.2^b^	735.9 ± 14.0^b^
ACVR2A	42.9 ± 0.4^ab^	143.9 ± 5.7	414.7 ± 22.0^ab^	615.2 ± 20.3^a^	705.7 ± 27.4^ab^
ACVR2B	41.2 ± 0.8^ab^	158.8 ± 5.6	454.2 ± 18.7^b^	703.4 ± 20.6^b^	779.1 ± 21.4^b^
*MSTN*-ACVR2A	37.7 ± 1.3^a^	147.8 ± 5.0	377.6 ± 13.1^a^	585.0 ± 19.0^a^	662.3 ± 25.0^a^
*MSTN*-ACVR2B	41.4 ± 0.9^ab^	152.0 ± 6.2	401.7 ± 19.5^ab^	640.7 ± 34.5^ab^	697.9 ± 31.2^a^
*MSTN*-ACVR2A-ACVR2B	40.4 ± 0.6^a^	159.3 ± 3.4	450.3 ± 16.3^b^	657.2 ± 32.6^b^	732.1 ± 32.8^b^
Control	39.0 ± 0.4^a^	143.2 ± 2.8	356.3 ± 6.3^a^	581.5 ± 8.8^a^	639.1 ± 10.3^a^

### Evaluation of muscle fibre

**Figure 7:**
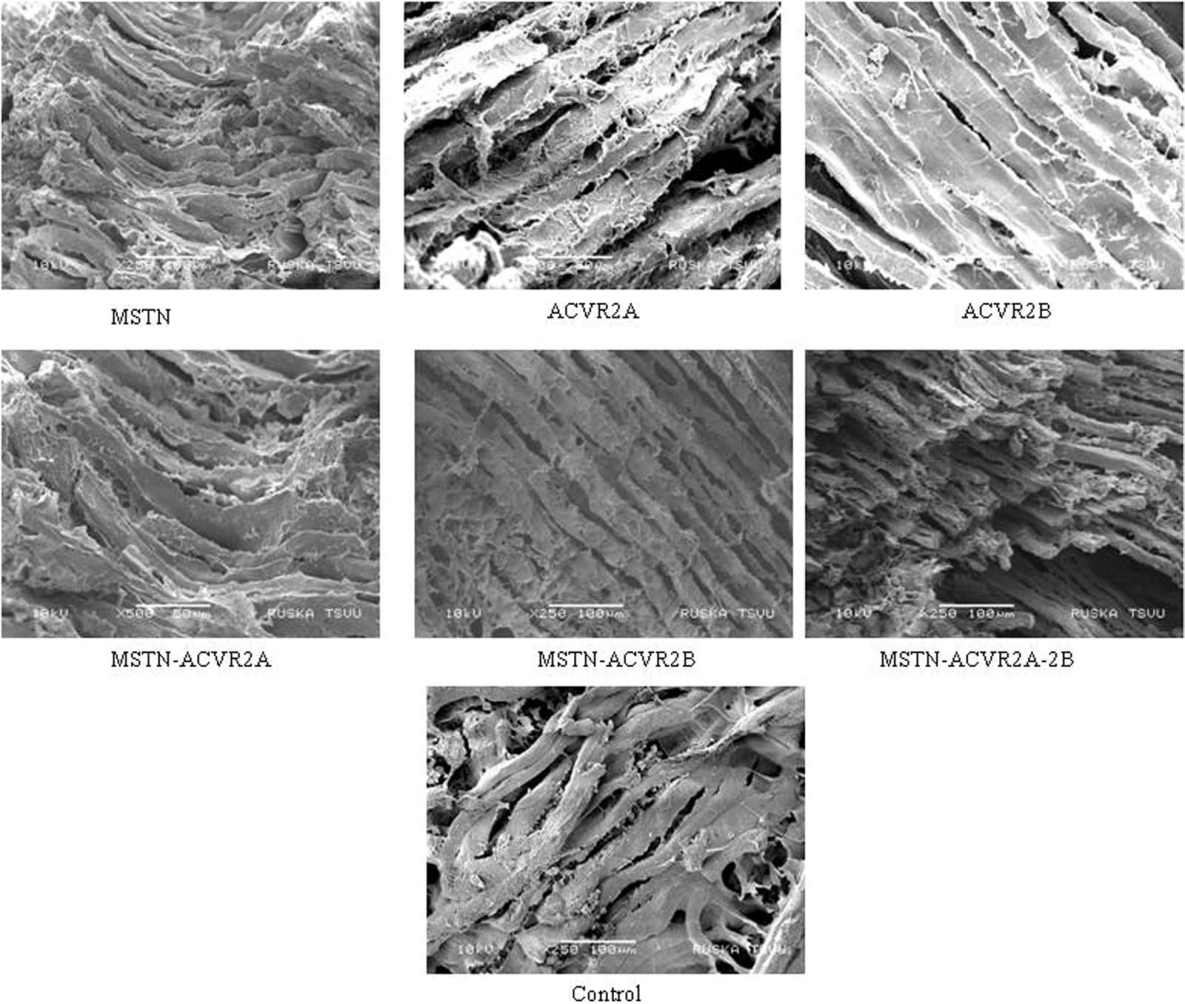
Electron microscopic view (SEM) of breast muscle fibres in the control and knock down birds for *MSTN*, ACVR2A and *ACVR2B* genes individually as well as in combination.

The electron microscopic views of muscle fibre of knock down and control birds have been presented in Fig. 7. The diameter of muscle fibres were significantly (P < 0.05) affected by silencing of *ACVR2B* and *MSTN* genes in transgenic chicken. The highest fibre diameter was observed in birds with silencing of *ACVR2B* gene (27.9 ± 0.8 µm) whereas the smallest fibre width was observed in control birds (16.1 ± 1.1 µm). In case of myofibril numbers in breast muscle, we observed significantly (P < 0.05) higher number of myofibrils in *ACVR2B*, *MSTN* and *MSTN*- *ACVR2A*- *ACVR2B* group as compared to control (Table 3). The highest number of myofibrils was found in *ACVR2B* group (4.1 ± 0.25). The silencing of *MSTN* and *MSTN*-*ACVR2A*-*ACVR2B* also showed significantly (P < 0.05) higher number of myofibrils (3.7 ± 0.22 and 3.4 ± 0.29, respectively) as compared to the control group (2.2 ± 0.23). We estimated correlation coefficient between body weights at different ages and muscle fibre number. The correlation between muscle fibre number and body weights at day old, 2^nd^, 4^th^, 5^th^ and 6^th^ week of age were 0.5, 0.8, 0.9, 0.9 and 0.9, respectively.

**Table 3:** Muscle fibre parameters in knock down and control chicken. Row-wise different superscripts indicate significance at P < 0.05.

Parameter	Knock down gene (s)						
	*MSTN*	ACVR2A	ACVR2B	*MSTN*-ACVR2A	*MSTN*-*ACVR2B*	*MSTN*-ACVR2A-*ACVR2B*	control
Muscle fibre size (µm)	19.9 ± 1.6^a^	26.1 ± 2.4^b^	27.9 ± 0.8^b^	26.1 ± 2.9^b^	27.7 ± 1.1^b^	26.2 ± 3.7^b^	16.1 ± 1.1^a^
Muscle fibre No.	3.7 ± 0.22^b^	2.8 ± 0.31^a^	4.1 ± 0.29^c^	2.5 ± 0.34^a^	2.6 ± 0.27^a^	3.4 ± 0.29^b^	2.2 ± 0.23^a^

### Evaluation of carcass traits

The carcass quality traits such as dressing%, leg muscle% and breast muscle% were significantly (P < 0.05) affected by silencing of *MSTN*, *ACVR2A* and *ACVR2B* genes (Table 4). The dressing% was significantly higher in birds with silencing of *ACVR2B*, *MSTN*-*ACVR2A*, *MSTN*-*ACVR2B* and *MSTN*-*ACVR2A*-*ACVR2B* genes as compared to the control birds. In case of breast muscle%, the highest magnitude was observed in birds with silencing of *MSTN*-*ACVR2B* gene (38.4 ± 2.9%). In case of leg muscle%, silencing of *ACVR2B* gene had the best (27 ± 0.5%) performance over other groups.

**Table 4:** Carcass characteristics in knock down and control chicken. Column-wise different superscripts indicate significance at P < 0.05.

Knock down gene (s)	Dressing%	Neck%	Breast%	Leg%	Spleen%	Bursa%	Back%	Heart%
*MSTN*	68.7 ± 6.8^a^	5.0 ± 0.3	25.0 ± 3.2^a^	27.1 ± 3.1^b^	0.2 ± 0.001	0.2 ± 0.001	30.5 ± 4.2	0.6 ± 0.006
ACVR2A	70.1 ± 3.2^a^	5.2 ± 0.3	31.0 ± 1.5^ab^	23.9 ± 0.9^ab^	0.2 ± 0.1	0.1 ± 0.001	25.7 ± 1.0	0.8 ± 0.1
*ACVR2B*	75.3 ± 1.8^b^	4.0 ± 0.3	31.4 ± 0.7^ab^	27.0 ± 0.5^b^	0.2 ± 0.001	0.2 ± 0.001	24.0 ± 0.3	0.6 ± 0.1
*MSTN*-ACVR2A	81.3 ± 3.0^b^	4.4 ± 0.3	33.9 ± 1.9^b^	22.3 ± 1.1^a^	0.2 ± 0.001	0.1 ± 0.001	23.5 ± 1.1	0.7 ± 0.0
*MSTN*-*ACVR2B*	77.6 ± 4.2^b^	3.9 ± 0.2	38.4 ± 2.9^b^	20.3 ± 1.7^a^	0.2 ± 0.001	0.1 ± 0.001	21.2 ± 1.5	0.6 ± 0.1
*MSTN*-ACVR2A-*ACVR2B*	80.4 ± 1.7^b^	3.5 ± 0.3	27.6 ± 2.2^a^	20.7 ± 1.3^a^	0.2 ± 0.001	0.1 ± 0.001	22.8 ± 0.9	0.5 ± 0.0
Control	69.5 ± 1.2^a^	3.3 ± 0.3	27.6 ± 2.1^a^	19.3 ± 1.3^a^	0.3 ± 0.001	0.2 ± 0.001	25.9 ± 1.7	0.7 ± 0.1

The serum cholesterol, triglycerides and HDL content were significantly (P < 0.05) affected by silencing of *ACVR2A*, *ACVR2B*, *MSTN*-*ACVR2A* and *MSTN*-*ACVR2B* genes (Table 5). In case of serum cholesterol content, the birds with silencing of *ACVR2B* gene had the lowest magnitude (72.1 ± 8.6 mgh/dL) over other groups of birds including control group (91.5 ± 3.6 mg/dL). In case of serum triglycerides, birds with silencing of *ACVR2A*, *ACVR2B* and *MSTN*-*ACVR2B* genes had significantly (P < 0.05) higher quantity (62.7 ± 16.8, 63.3 ± 28.6 and 55.8 ± 12.6mg/dL, respectively) in serum over the control group of birds (30.6 ± 4.4 mg/dL). In case of HDL, birds with silencing of *ACVR2B* and *MSTN*-*ACVR2A* genes showed significantly (P < 0.05) higher quantity (46.8 ± 3.1 and 50.5 ± 4.0 mg/dL, respectively) than the control group of birds (20.0 ± 3.4 mg/dL).

**Table 5:** Serum biochemical parameters in knock down and control chicken. Column-wise different superscripts indicate significance at P < 0.05.

Knock down gene (s)	Cholesterol (mg/dL)	Triglycerides (mg/dL)	HDL (mg/dL)
*MSTN*	97.5 ± 20.1^b^	22.8 ± 7.8^a^	11.2 ± 3.4^a^
ACVR2A	98.5 ± 19.1^b^	62.7 ± 16.8^b^	22.9 ± 5.2^a^
*ACVR2B*	72.1 ± 8.6^a^	63.3 ± 28.6^b^	46.8 ± 3.1^b^
*MSTN*-ACVR2A	131.8 ± 9.2^c^	26.7 ± 3.9^a^	50.5 ± 4.0^b^
*MSTN*-*ACVR2B*	97.1 ± 13.0^b^	55.8 ± 12.6^b^	24.7 ± 7.0^a^
*MSTN*-ACVR2A-*ACVR2B*	86.5 ± 6.3^b^	37.6 ± 11.9^a^	25.5 ± 6.2^a^
Control	91.5 ± 3.6^b^	30.6 ± 4.4^a^	20.0 ± 3.4^a^

### Evaluation of blood cell profile

The blood cell profile was explored in knock down and control birds where significantly different total RBC and WBC count were observed among the knock down and control groups (Table 2S). The significantly higher number of RBC was found in birds silenced with *ACVR2B* (5.35 ± 0.15 million/ml) and *MSTN*-*ACVR2A*-*ACVR2B* (5.09 ± 0.23million/ml) genes. In all the groups of knock down birds except for *MSTN*, the total RBC was found to be higher than control group. In case of WBC count, the number was significantly reduced in all knock down birds as compared to the control one. However, the highest reduction over control group was detected in the birds of *ACVR2A* silencing (5884 ± 444 *vs* 9459 ± 898). The birds of *MSTN*-*ACVR2A* and *MSTN*-*ACVR2A*-*ACVR2B* groups did not show significant changes of total WBC count as compared to that of the control group of birds. Other blood cells did not show any significant change between knock down and control groups.

## Discussion

The transgenic knock down chicken was produced by employing sperm mediated gene transfer method where the average success rate of 40.5% was quite encouraging. In earlier studies, we standardized the techniques of production of transgenic knock down chicken, where we observed that the sperm mediated method was quite efficient with variable success rate in the range of 30.5 to 40.9% to produce knock down birds^31^. In another study, authors reported sperm transfection assisted gene editing (STAGE) an alternative technique to produce knock out or transgenic chicken^32^. However, the present sperm mediated method has been successful to develop knock down/transgenic chicken for *MSTN* and its receptors. For each gene, 2 best shRNA molecules tested previously in cell culture were used *in vivo* to produce knock down transgenic chicken^33–35^. The silencing of three genes namely, *MSTN* and its receptors, *ACVR2A* and *ACVR2B* were analysed separately as well as in different combinations such as *MSTN*-*ACVR2A*, *MSTN*-*ACVR2B* and *MSTN*-*ACVR2A*-*ACVR2B*. The knock down birds did not show any anatomical changes, but we found 7.1 to 37.5% dead chicks in all knock down groups except *MSTN* knock down group. The highest death percentage was observed in knocking down of combination of all three genes. This may be due to improper muscular development and physiological inefficiency of embryo on account of knocking down of *MSTN*, *ACVR2A* and *ACVR2B* genes. However, healthy knock down chicks were sacrificed at 6 weeks of age on account of collection of tissues and assessing carcass quality traits.

Important physiological parameters such as blood cell profiles reveals that the birds of *ACVR2B* group had 25.5% higher number of RBC than that of the control group, which may be due to mitigating growing requirement of cellular metabolism in relatively large sized body of transgenic chicken compared to the control birds. We suggest that silencing of *MSTN* alone cannot increase the number of RBC but in presence of silencing of *ACVR2A* and *ACVR2B*, the number of total RBC have been increased to meet the body requirement such as supply of oxygen to the cells. The other blood cell such as WBC count was also influenced by gene silencing. The total WBC count was significantly decreased in single knock down of *MSTN*, *ACVR2A* and *ACVR2B* genes as compared to the control group. It is known that WBCs are important in conferring immunity to the animals^36^. Lowering the number of WBCs on account of gene knock down may have deleterious effect in sustaining immunity in the birds.

We compared the growth performance of the chicks derived from knock down and control groups. The body weights at different ages during juvenile stages were significantly affected by silencing of *MSTN*, *ACVR2A* and *ACVR2B* genes in chicken. At day old age, the birds with silencing of *MSTN* gene had the highest body weight in knock down group with 17.6% superiority over the control group of birds. At 35 and 42 days of age, silencing of *ACVR2B* gene took upper hand having the highest body weights with 20.8 and 21.9% superiority, respectively over the control group. In addition, carcass traits which are very important in chicken were also analyzed in transgenic and non-transgenic control birds. The dressing% which has been the major indicator of carcass quality was found as significantly lower in control group of birds as compared to *ACVR2B*, *MSTN*-*ACVR2A*, *MSTN*-*ACVR2B* and *MSTN*-*ACVR2A*-*ACVR2B* knock down birds where the trend of dressing % was *MSTN*-*ACVR2A* > *MSTN*-*ACVR2A*-*ACVR2B* > *MSTN*-*ACVR2B* > *ACVR2B* > *MSTN*. The *MSTN*-*ACVR2A*, *MSTN*-*ACVR2A*-*ACVR2B*, *MSTN*-*ACVR2B* and *ACVR2B* silenced birds had 16.9%, 15.6%, 11.6% and 8.3% superiority of dressing% over the control group birds.

Other important carcass trait such as breast muscle and leg muscle% were also in higher side in knock down chicken over the control birds. The significantly higher magnitudes were observed in *MSTN*-*ACVR2B* (38.4 ± 2.9%) and *MSTN*-*ACVR2A* (33.9 ± 1.9%) knock down birds for breast muscle and *MSTN* (27.1 ± 3.1%) and *ACVR2B* (27.0 ± 0.5%) knock down birds for leg muscle over control group of birds (27.6 ± 2.1 and 19.3 ± 1.3%, respectively). The similar trend of muscular hypertrophy was observed in *MSTN* knock down in Zebra fish^37^, catfish^38^ and human^39^ revealing importance gene silencing for *MSTN* for enhancing growth.

In meat animals, another major factor for meat consumption is serum cholesterol which compels us to eat chicken products in limited quantity. Higher serum cholesterol will make chicken produce rich in cholesterol content, which is the most undesired component of chicken egg and meat. Cholesterol is the most important predisposing factor for cardiac ailment. In this study, the serum cholesterol content was significantly lowest in *ACVR2B* knock down chicken over the control group emphasizing acceptability of poultry produce developed through this technique. The HDL content in serum is very important as HDL has been the good lipid required in the body for maintaining different physiological functions such as causing reverse cholesterol transport from the periphery to the liver and controls pleiotropic effects on inflammation, haemostasis and apoptosis leading to minimizing coronary heart diseases in human^40^, muscular dystrophy and type II diabetes^41^. In our study, we have found significantly (P < 0.05) higher serum HDL content in the birds of *ACVR2B* and *MSTN*-*ACVR2A* group as compared to the control one. The *ACVR2B* and *MSTN*- *ACVR2A* group had 134% and 152.5% higher HDL content in serum, respectively over the control birds. As the HDL content was high and serum cholesterol content was low in birds with *ACVR2B* silencing, it may be considered that meat of *ACVR2B* silenced birds may be of good quality with nutritionally best suited to the consumers with heart and high blood pressure ailments. Thus, the enhancement of quality of chicken meat can be possible through silencing of these genes both individually and in combination. We suggest that out of these three negative factors for controlling growth, single knock down for *ACVR2B* gene might be the best approach for improving growth in chicken. By knocking down only one factor, *ACVR2B* is sufficient to obtain optimum results as *MSTN* would not find its receptor to act negatively on growth. Thus, we can suggest that RNAi technology may be applied for augmenting production performance of animals and treatment of diseases in human and animals^42^.

It is concluded that single knock down of *MSTN* and its receptors (*ACVR2A* and *ACVR2B*) had potential to enhance body weight as compared to the combinations of *MSTN* and its receptors. These novel findings may help to utilize this technique for enhancing body mass in other food animals and to explore this technique in curing muscular decaying diseases in human being.

## Methods

### Animals

The broiler based PD-1 chicken line maintained at the Institute farm, ICAR-Directorate of Poultry Research, Hyderabad, India was included in the present study. A total of 280 fertile eggs were collected from 134 hens inseminated with the pooled semen prepared by collecting from 35 cocks. The experimental design has been detailed in Table 1. The whole study has been approved by the Institute Animal Ethics committee (IAEC) and Institute Biosafety Committee (IBSC) of ICAR-Directorate of Poultry Research, Hyderabad, India. The work was also noted by the RCGM, Dept. of Biotechnology, Govt. of India and the bio-safety guidelines of RCGM were also followed during the experiment. The birds were sacrificed following the standard protocol of cervical dislocation approved by the IAEC committee of the Institute. The animal welfare measures like *ad lib* feeding, watering and management of the hatched birds were taken care off by providing required space during the experiment.

### Designing and cloning of shRNA oligos

**Figure 8:**
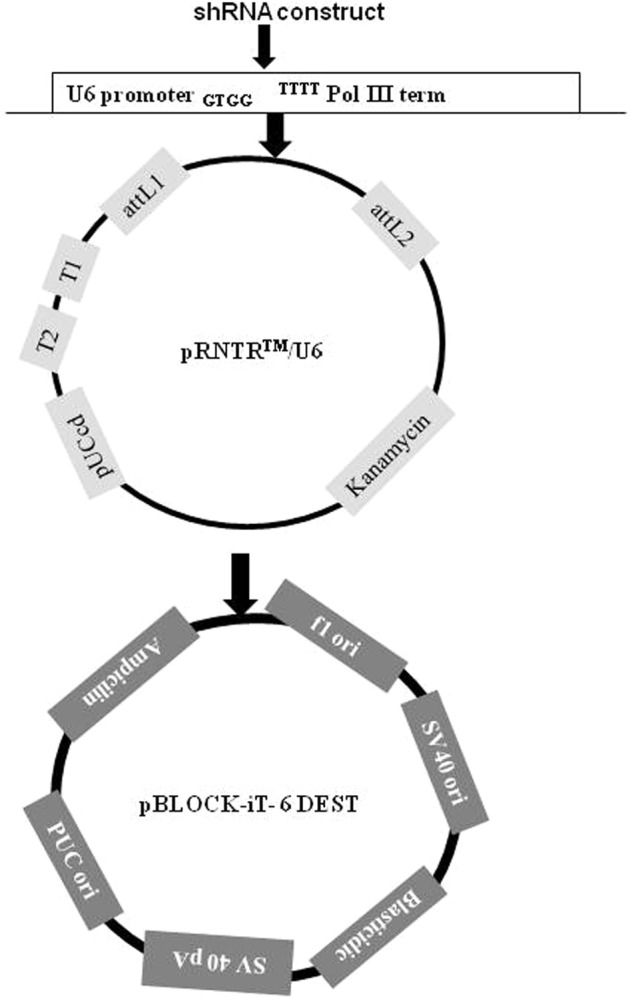
Schematic diagram of maps of two vectors (pRNTR^TM^/U6 and pBLOCK-iT- 6 DEST).

The shRNA molecules were designed from the coding sequence of chicken *MSTN* (Accession No. AF346599), *ACVR2A* (Accession No. NM_205367.1) and *ACVR2B* genes (Accession No. NC_204317) with Invitrogen Block-iT RNAi designer programme (Table 6). Two best shRNA molecules were identified based on our previous study conducted under *in vitro* cell culture system for *MSTN*^31^, *ACVR2A*^32^ and *ACVR2B* genes^33^. In this study, BLOCK-iT U6 RNAi Entry Vector (Invitrogen) and pBLOCK-iT DEST6 vector (Invitrogen) were used to generate permanent expression clones. Earlier, the pBLOCK-iT Dest vector was also reported to be used for stable RNA interference against CXCR7 gene^43^. The pBLOCK-iT™6-DEST vector is promoterless Gateway® destination vector into which the RNAi cassette is transferred. The DEST vector allows stable expression of short hairpin RNA (shRNA) in dividing eukaryotic cells for RNAi analysis. Double-stranded oligo encoding an shRNA to the target gene was cloned in U6 promoter guided pENTR™/U6 vector to prepare RNAi cassette. The entire RNAi cassette is recombined to the pBLOCK-iT™-DEST vector to generate the expression clone so that shRNA molecules are synthesized in the body (Fig. 8).

**Table 6:** shRNA molecules against *MSTN*, ACVR2A and *ACVR2B* genes transfected for production of knock down chicken.

Gene	Molecule name	shRNA molecules (5′-3′)	NCBI Accession No.	Location in the gene	Reference
*MSTN*	shRNA3	CACCGACTGTGATGAGCACTCAACGCGAACGTTGAGTGCTCATCACAGTC	AF346599	298	Bhattacharya *et al*.^33^
		AAAAGACTGTGATGAGCACTCAACGTTCGCGTTGAGTGCTCATCACAGTC			
	shRNA4	CACCGCTCCGGAGAATGTGAATTTGCGAACAAATTCACATTCTCCGGAGC		811	
		AAAAGCTCCGGAGAATGTGAATTTGTTCGCAAATTCACATTCTCCGGAGC			
ACVR2A	shRNA1	CACCGCAGTTGCTAGAGATCAAAGCCGAAGCTTTGATCTCTAGCAACTGC	NM_205367.1	89	Satheesh *et al*.^34^
		AAAAGCAGTTGCTAGAGATCAAAGCTTCGGCTTTGATCTCTAGCAACTGC			
	shRNA2	CACCGGATGTACAGACATCACAAGCCGAAGCTTGTGATGTCTGTACATCC		476	
		AAAAGGATGTACAGACATCACAAGCTTCGGCTTGTGATGTCTGTACATCC			
*ACVR2B*	shRNA1	CACCGCAACTACTGCAATGAGAAATCGAAATTTCTCATTGCAGTAGTTGC	NC_204317	317	Guru Vishnu (2016)
		AAAACAACTACTGCAATGAGAAATTTCGATTTCTCATTGCAGTAGTTGC			
	shRNA3	CACCGGTGGGAACAAGGAGGTATATCGAAATATACCTCCTTGTTCCCACC		836	
		AAAAGTGGGAACAAGGAGGTATATTTCGATATACCTCCTTGTTCCCACC

### Sperm transfection with shRNA molecules

A total of 35 cocks of PD-1 broiler chicken line were randomly selected for collection of semen. The semen collected from all the animals were pooled and centrifuged at 500 g for 10 min to remove seminal plasma. The sperms were washed with HT ‘High Temperature’ buffer (NaCl-0.8 g, TES buffer-1.37 g and glucose-0.69 g) for three times. The total number of sperm was counted in the Neubeur chamber. The sperms were transfected with 10 µg shRNA of myostatin, *ACVR2A* and *ACVR2B* clones by electroporation with Gene Pulser (Biorad) at 160 mV for 25 milisecond for 1 pulse. A volume of 0.25 ml HT buffer containing about 100 Million sperm was inseminated to each hen and accordingly, the total quantity of sperm was divided into 6 knock down groups and 1 control group (Only electrical impulse without DNA was given to the sperm before insemination). Accordingly, the sperm allotted to each group was transfectd separately with by electroporation. The transfected sperms were inseminated to each hen under each treatment group. The insemination with same protocol was repeated on the consecutive days and from 3^rd^ day onwards, eggs were collected and kept at 4 °C temperature till incubation. The eggs were incubated at the incubator for 18 days at 98–100 °F with 78–80% relative humidity and turning 6 times a day. Then, eggs were candled on 19^th^ day and the fertile eggs were further kept in the hatcher for 3 days at 98–100 °F with 78–80% relative humidity. The chicks were hatched and and all chicks were marked with wing band. The chicks were maintained in the brooder house upto 6 weeks of age by providing *ad lib* feeding, watering and lighting to warm the house.

### Screening of positive knock-down birds by PCR and Southern blotting

Blood samples were collected from all the birds under study alongwith the control group. Genomic DNA was isolated following standard protocol^44^. The genomic DNA was used for PCR and Southern blotting. For PCR, a pair of primers (DESTFQ: ACGTCGACGGATCGGGACGAGATC DESTRQ: CGCTAGGGCGCTGGCAAGTG) were designed from the DEST vector for amplification of 550 bp fragment of the vector encompassing 50 bp shRNA molecules of the genes. Through PCR, the positive birds were screened first and then, all the positive birds were also subjected to Southern blotting by digesting with *Pst*I restriction enzyme for confirmation. Probes were prepared from DEST vector, which were lebeled with biotin-streptavidin conjugated to alkalin phosphatase and detected with NBT/BCIP for spot hybridization.

### Real time PCR

For real time PCR, total RNA was isolated from the breast muscle tissues of birds of the treatment as well as and control groups using Trizol according to the Manufacturer’s instruction (Sigma). The total RNA was reverse transcribed with oligo dT primer to synthesize single stranded cDNA. The real time PCR of the respective treatment and control groups was performed in duplicate for myostatin, *ACVR2A*, *ACVR2B* and *GAPDH* genes with SYBR Green chemistry. The pairs of primers designed for *MSTN* (Accession No.AF346599), *ACVR2A* (Accession No. NM_205367.1) and *ACVR2B* (Accession No. NC_204317) and GAPDH (Accession No. NM_204305.1) genes for real time PCR were MYTQF: GCA AAA GCT AGC AGT CTA TG and MYTQR: TCC GTC TTT TTC AGC GTT CT for myostatin, 5′-GATCCTGGACCACCACCGC-3′ and 5′-CTGGATAGGGAATATTTTGACTG-3′ for *ACVR2A*, F: 5′-AGGGCAACTACTGCAATGAG-3′ and R:5′-CACTGACAGGACAGCGATG-3′ for *ACVR2B* and QGAPDHF: 5′-CTGCCGTCCTCTCTGGC-3′ and QGAPDHR: 5′-GACAGTGCCCTTGAAGTGT-3′ for GAPDH genes. The GAPDH was used as reference gene. The amplification and comparative analysis of mRNA expression was performed by calculating 40-Δct in both knock down and control groups according to the method mentioned in the literature^45^, where, ∆*C*_*t*_ = Average C_t_ of target gene – Average C_t_ of reference gene.

Fold change of expression = 2^−∆∆Ct ^^46^, where

$${\rm{\Delta }}{{C}}_{{t}}={\rm{Average}}\,{{C}}_{{t}}\,{\rm{of}}\,{\rm{target}}\,{\rm{gene}}-{\rm{Average}}\,{{C}}_{{t}}\,{\rm{of}}\,{\rm{reference}}\,{\rm{gene}}$$

$${\rm{\Delta }}{\rm{\Delta }}{{C}}_{{t}}=\mathrm{Average}\,{\rm{\Delta }}{{C}}_{{t}}\,{\rm{of}}\,{\rm{target}}\,{\rm{gene}}-\mathrm{Average}\,{\rm{\Delta }}{{C}}_{{t}}\,{\rm{of}}\,{\rm{calibrator}}\,{\rm{gene}}$$

### Western blotting

The birds of all the groups at the age of 6 weeks were slaughtered by cervical dislocation method approved by the Institute Animal Ethics Committee of ICAR-Directorate of Poultry Research, Hyderabad, India. The breast muscle tissues of all the birds were collected and the crude protein was prepared from the tissues. The protein samples were electrophoresed in Tris-Glysin-SDS buffer (pH 8.3) at 60 V in 12% polyacrylamide gels of which one set of gels were stained with coomassie brilliant blue for visualization of resolved protein bands and another set of gels were used to transfer proteins onto PVDF membrane in the semidry transfer apparatus (Biorad) at 100 V for 1 hour. Then, the membrane was treated with anti rat IgG-HRP conjugate (Sigma) diluted to 1:1000 in TBS Tween buffer and visualized by treatment with DAB substrate using standard protocol^45^.

### Elisa

The ELISA of serum samples of the experimental birds were carried out to detect the level of MSTN, ACVR2A and ACVR2B proteins in the knock down and control birds. Detection of myostatin, ACVR2A and ACVR2B are based on standard sandwich enzyme-linked immuno-sorbent assay technology. An antibody specific for MSTN or ACVR2A or ACVR2B had been pre-coated onto a 96-well plate (12 × 8 Well Strips) and blocked. Standards or test samples are added to the wells, incubated and removed. HRP detector antibody specific for MSTN or ACVR2A or ACVR2B was added, incubated and followed by washing. HRP-Peroxidase Conjugate was then added, incubated and unbound conjugate was washed away. An enzymatic reaction was produced through the addition of TMB substrate which was catalyzed by HRP generating a blue color product that changed yellow after adding acidic stop solution. The density of yellow coloration read by absorbance at 450 nm in ELISA reader was quantitatively proportional to the amount of sample MSTN or ACVR2A or ACVR2B captured in the well. The absorbance values for MSTN, ACVR2A and ACVR2B protein content in the serum of knock down and control group of birds estimated through ELISA were compared between knock down and control groups, and between titres within each group following LSD test (SPSS 20.0 software).

### Growth and carcass traits

All the experimental birds including control were included in the study for measuring body weights at day1 and 2^nd^, 4^th^, and 6^th^ week of age. The growth rates between day1 to day14, day15 to day28 and day29 to day42 were calculated for all the birds. Feed efficiency of all the birds were calculated by amount of feed consumed for attaining 1 kg body weight at 6 weeks of age. All the experimental birds were sacrificed by cervical dislocation. Different slaughter parameters such as carcass weight, dressing%, breast%, leg%, back%, wing%, neck% and heart% were measured.

### Blood cell profiles and biochemical parameters

Blood samples were collected from the knock-down, scrambled and control birds. The blood cells were counted by hemocytometer for all the samples. For RBC counting, blood was taken upto 0.5 mark in the RBC pipette and then, Hyaem’s fluid was sucked upto 101 mark in that pipette. Then, blood and Hyaem’s fluid was mixed properly and initial few drops of blood mixture was thrown out. Then, the blood mixture was charged in the Neubeur chamber of the Haemocytometer and RBC was counted in the chamber with 40X magnification under microscope. Total RBC concentration was calculated following the standard formula.

$${\rm{Total}}\,{\rm{RBC}}/{{\rm{mm}}}^{3}=({\rm{Number}}\,{\rm{of}}\,{\rm{RBC}}\,{\rm{counted}}\times {\rm{Dilution}}\,{\rm{factor}}\times {\rm{Depth}}\,{\rm{factor}})/{\rm{No}}{\rm{.}}\,{\rm{of}}\,{\rm{chambers}}\,{\rm{counted}}$$

For total WBC counting, blood was taken upto 0.5 mark in the WBC pipette and then, Truck’s fluid (WBC dilution fluid) was sucked upto 11 mark in that pipette. Then, blood and Truck’s fluid was mixed properly and initial 3 to 4 drops of mixture was thrown out. Then, the blood mixture was charged in the Neubeur chamber of the Haemocytometer and WBC was counted in the chamber with 10X magnification under microscope. Total WBC concentration was calculated following the standard formula.

$${\rm{Total}}\,{\rm{WBC}}/{{\rm{mm}}}^{3}=({\rm{Number}}\,{\rm{of}}\,{\rm{WBC}}\,{\rm{counted}}\times {\rm{Dilution}}\,{\rm{factor}}\times {\rm{Depth}}\,{\rm{factor}})/{\rm{No}}{\rm{.}}\,{\rm{of}}\,{\rm{chambers}}\,{\rm{counted}}$$

For differential count, blood smear was prepared on a glass slide and fixed with methanol. The slide was stained first with Field stain-B (Eosin- 1 g; Disodium hydrogen phosphate-5g; Potassium dihydrogen phosphate-6.25 g; Distilled water-500 ml) and then, with Field stain-A (Azrure I- 0.5 g; Disodium hydrogen phosphate-5g; Potassium dihydrogen phosphate-6.25 g; Methylene blue- 0.8 g; Distilled water-500 ml). The slide was washed in water, dried and observed under the Microscope to visualize the WBCs (Neutrophil, Eosinophil, Basophil, Lymphocyte and Monocyte), where cytoplasm took acidic stain and nucleus took basic stain.

The blood samples without anti-coagulant were used to collect serum of all the birds. The serum samples were used to estimate triglyceride, total cholesterol and HDL in Blood analyzer with the analysis kit (Jeev Diagnostics Pvt. Ltd, India) following Manufacturer’s protocol.

### Electron Microscopy

Breast muscle sample of two birds from each experimental group including control were fixed in 2.5% gluteradehyde in 0.1 M phosphate buffer for 30 minutes (ph 7.2) and were mounted over cover glass placed on the double sided carbon conductivity tape of the aluminum stubs. After fixing, cover slips were put on 1% aqueous osmium tetraoxide fumes for 30 mints, and exposed to 80% and 100% alcohol fumes each 15 minutes and dried under vacuum desiccation for 15 minutes. A thin layer of gold coating over the samples were done by using an automated sputter coater (Model-JEOL JFC-1600) for 3 minutes and scanned under Scanning Electron Microscope (SEM-Model:JOEL-JSM 5600) at required magnification as per the standard procedure at RUSKA Lab, College of Veterinary Science, PVNRTVU, Rajendranagar, Hyderabad, India^47^. The muscle fibre diameter (in millimeter unit) was measured at different location of the fibre with Vernier calipers and then, divided by the magnification observed under the microscope to arrive the average diameter of the muscle fibre. The diemeter estimation was also performed at RUSKA Lab, College of Veterinary Science, PVNRTVU, Rajendranagar, Hyderabad, India.

### Immuno-histochemistry

The breast muscle tissues were collected from knock-down and control birds. The tissues were kept in formalin (10%) overnight. Then, the tissues were cleaned with running tap water water. The tissues were treated with Xyline and put in paraffin wax at −10 °C. On next day, slides (6 µm) were prepared using microtome machine (Medimeas: Model No. MRM-ST) and kept for drying at 40 °C for 2 to 3 hours. The slides were stained with Haematoxyline-Eosine stain. The slides were mounted with DPX and examined under microscope for counting muscle fibres.

### Statistical analysis

The effect of shRNA molecules of *MSTN*, *ACVR2A* and *ACVR2B* genes on growth traits, carcass traits, blood profiles, serum biochemical parameters, and number as well as diameter of muscle fibres were analysed by GLM procedure with SPSS20.0 software. The shRNA class and sex of the birds were used as fixed effects. The model used for analyzing effects was Y = µ + R_i_ + S_j_ + E_ijk_

Where, Y = Trait; µ = Overall mean; Ri = Fixed effect of i^th^ shRNA molecule; S_j_ = Fixed effect of j^th^ sex and E_ijk_ = k^th^ residual effect

The Duncan’s multiple range test (DMRT) was performed to determine the effect of each group. The correlation coefficient between number of muscle fibres and body weights at different ages were estimated following standard statistical method (SPSS20.0).

## Supplementary information

Dataset 1
