# Supplementary material for: Comparative analysis of silencing expression of myostatin (MSTN) and its two receptors (ACVR2A and ACVR2B) genes affecting growth traits in knock down chicken
Source: Sci Rep. 2019 May 24;9:7789. doi: 10.1038/s41598-019-44217-z (PMC6534594; doi:10.1038/s41598-019-44217-z)
Supplement: Supplementary file 1 — Dataset 1 [file 41598_2019_44217_MOESM1_ESM.docx]

**Supplementary files**

**Comparative analysis of silencing expression of myostatin (*MSTN*) and its two receptors (*ACVR2A* and *ACVR2B*) genes affecting growth traits in knock down chicken**

T.K. Bhattacharya, Renu Shukla, R.N. Chatterjee and S.K. Bhanja

Table 1S. Protein concentration (Absorbance at OD_450_) in serum detected by Sandwich ELISA in knock down and control birds. Within row and between rows (Knock down for specific gene and control), the superscripts a, b, c & d denote significance at P<0.05 for MSTN. Within row and between rows (Knock down for specific gene and control), the superscripts A & B denote significance at P<0.05 for ACVR2A. Within row and between rows (Knock down for specific gene and control), the superscripts X, Y & Z denote significance at P<0.05 for ACVR2B content in different groups.

| **Knock down gene (s)** | **Titre** | | | | | | |
| --- | --- | --- | --- | --- | --- | --- | --- |
|  | **1:250** | **1:500** | **1:1000** | **1:1500** | **1:2000** | **1:4000** | **1:8000** |
| MSTN | 0.173^b^ | 0.178 ^b^ | 0.147 ^b^ | 0.134 ^b^ | 0.126 ^b^ | 0.075 ^a^ | 0.032 ^a^ |
| ACVR2A | 0.203^B^ | 0.197 ^B^ | 0.184 ^B^ | 0.167 ^B^ | 0.142 ^B^ | 0.082 ^A^ | 0.037 ^A^ |
| ACVR2B | 0.214^Y^ | 0.201 ^Y^ | 0.192 ^Y^ | 0.178 ^Y^ | 0.153 ^Y^ | 0.091 ^X^ | 0.043 ^X^ |
| MSTN-ACVR2A | 0.163 ^b^ (MSTN) 0.178 ^B^ (ACVR2A) | 0.154 ^b^ (MSTN) 0.171 ^B^ (ACVR2A) | 0.142 ^b^ (MSTN  0.163 ^B^ (ACVR2A) | 0.129 ^b^ (MSTN)  0.159 ^B^ (AC VR2A) | 0.121 ^b^ (MSTN)  0.146 ^B^ (ACVR2A) | 0.072 ^a^ (MSTN) 0.079 ^A^ (ACVR2A) | 0.037 ^a^ (MSTN)  0.035 ^A^ (ACVR2A) |
| MSTN-ACVR2B | 0.172 ^b^ (MSTN) 0.181 ^Y^ (ACVR2B) | 0.169 ^b^ (MSRN) 0.177 ^Y^ (ACVR2B) | 0.149 ^b^ (MSTN)  0.168 ^Y^ (ACVR2B) | 0.141 ^b^ (MSTN)  0.159 ^Y^ (ACVR2B) | 0.119 ^b^ (MSTN)  0.149 ^Y^ (ACVR2B) | 0.074 ^a^ (MSTN) 0.081 ^X^ (ACVR2B) | 0.039 ^a^ (MSTN)  0.041 ^X^ (ACVR2B) |
| MSTN-ACVR2A-2B | 0.205 ^b^ (MSTN) 0.174 ^B^ (ACVR2A) 0.181 ^Y^ (ACVR2B) | 0.209 ^b^ (MSTN) 0.169 ^B^ (ACVR2A) 0.171 ^Y^ (ACVR2B) | 0.187 ^b^ (MSTN)  0.169 ^B^ (ACVR2A) 0.169 ^Y^ (ACVR2B) | 0.164 ^b^ (MSTN)  0.168 ^B^ (ACVR2A)  0.162 ^Y^ (ACVR2B) | 0.148 ^b^ (MSTN) 0.154 ^B^ (ACVR2A) 0.157 ^Y^ (ACVR2B) | 0.073 ^a^ (MSTN)  0.083 ^A^ (ACVR2A)  0.087 ^X^ (ACVR2B) | 0.031 ^a^ (MSTN)  0.036 ^A^ (ACVR2A) 0.039 ^X^ (ACVR2B) |
| Control (Negative) | 0.429 ^d^ (MSTN) 0.397 ^C^ (ACVR2A) 0.401 ^Z^ (ACVR2B) | 0.391^d^ (MSTN)  0.387 ^C^ (ACVR2A)  0.391 ^Z^ (ACVR2B) | 0.367 ^d^ (MSTN)  0.373 ^C^ (ACVR2A) 0.369 ^Z^ (ACVR2B) | 0.347 ^d^ (MSTN) 0.359 ^C^ (ACVR2A) 0.342 ^Z^ (ACVR2B) | 0.297 ^c^ (MSTN) 0.311 ^C^ (ACVR2A) 0.325 ^Z^ (ACVR2B) | 0.277 ^c^ (MSTN) 0.296 ^BC^ (ACVR2A) 0.285 ^Y^ (ACVR2B) | 0.211 ^c^  (MSTN)  0.215 ^B^ (ACVR2A) 0.207 ^Y^ (ACVR2B) |

Table 2S. Blood cell profile in knock down and control group of chicken. Column-wise different superscripts indicate significance at P<0.05.

| **Knock down gene (s)** | **RBC (Million)** | **WBC** | **Lymphocyte %** | **Monocyte %** | **Heterophil %** | **Basophil %** | **Eosinophil %** |
| --- | --- | --- | --- | --- | --- | --- | --- |
| MSTN | 3.66 ± 0.12 ^a^ | 6920 ±679 ^a^ | 73.7 ±4.71 | 2.79 ±0.31 | 19.4 ±1.60 | 1.69 ±0.33 | 1.74 ±0.21 |
| ACVR2A | 4.60 ± 0.37 ^ab^ | 5884 ± 444 ^a^ | 69.5 ± 5.37 | 3.4 ± 0.41 | 21.2 ± 1.43 | 2.57 ± 0.17 | 2.71 ± 0.31 |
| ACVR2B | 5.35 ± 0.15 ^b^ | 6828 ± 797 ^a^ | 70.9 ± 3.23 | 3.4 ± 0.30 | 20.0 ± 1.12 | 2.53 ± 0.41 | 3.00 ± 0.30 |
| MSTN-ACVR2A | 4.70 ± 0.19 ^ab^ | 8584 ± 609 ^b^ | 71.9 ± 5.00 | 4.4 ± 0.30 | 17.7 ± 0.91 | 2.90 ± 0.36 | 2.83 ± 0.30 |
| MSTN-ACVR2B | 4.98 ± 0.22 ^ab^ | 7675 ± 334 ^a^ | 71.3 ± 4.72 | 3.8 ± 0.42 | 18.0 ± 1.52 | 3.11 ± 0.24 | 2.91 ± 0.30 |
| MSTN-ACVR2A-ACVR2B | 5.09 ± 0.23 ^b^ | 9162 ± 591 ^b^ | 71.3 ± 4.41 | 3.9 ± 0.20 | 18.6 ± 0.93 | 3.60 ± 0.31 | 2.95 ± 0.40 |
| Control | 4.29 ± 0.14 ^a^ | 9459 ± 898 ^b^ | 69.5 ± 3.35 | 4.3 ± 0.20 | 20.5 ± 1.16 | 3.20 ± 0.30 | 2.43 ± 0.31 |
